# The Spectrum of Mutations of Homocystinuria in the MENA Region

**DOI:** 10.3390/genes11030330

**Published:** 2020-03-20

**Authors:** Duaa W. Al-Sadeq, Gheyath K. Nasrallah

**Affiliations:** 1 Biomedical Research Center, Qatar University, P.O. Box 2713 Doha, Qatar; 2 College of Medicine, Member of QU Health, Qatar University, P.O. Box 2713 Doha, Qatar; 3 Department of Biomedical Science, College of Health Sciences, Qatar University, P.O. Box 2713 Doha, Qatar

**Keywords:** homocystinuria, cystathionine β-synthase, mutations, MENA

## Abstract

Homocystinuria is an inborn error of metabolism due to the deficiency in cystathionine beta-synthase (CBS) enzyme activity. It leads to the elevation of both homocysteine and methionine levels in the blood and urine. Consequently, this build-up could lead to several complications such as nearsightedness, dislocated eye lenses, a variety of psychiatric and behavioral disorders, as well as vascular system complications. The prevalence of homocystinuria is around 1/200,000 births worldwide. However, its prevalence in the Gulf region, notably Qatar, is exceptionally high and reached 1:1800. To date, more than 191 pathogenic *CBS* mutations have been documented. The majority of these mutations were identified in Caucasians of European ancestry, whereas only a few mutations from African-Americans or Asians were reported. Approximately 87% of all *CBS* mutations are missense and do not target the CBS catalytic site, but rather result in unstable misfolded proteins lacking the normal biological function, designating them for degradation. The early detection of homocystinuria along with low protein and methionine-restricted diet is the best treatment approach for all types of homocystinuria patients. Yet, less than 50% of affected individuals show a significant reduction in plasma homocysteine levels after treatment. Patients who fail to lower the elevated homocysteine levels, through high protein-restricted diet or by B6 and folic acid supplements, are at higher risk for cardiovascular diseases, neurodegenerative diseases, neural tube defects, and other severe clinical complications. This review aims to examine the mutations spectrum of the *CBS* gene, the disease management, as well as the current and potential treatment approaches with a greater emphasis on studies reported in the Middle East and North Africa (MENA) region.

## 1. Introduction

Inborn errors of metabolism represent a large group of genetic disorders that result from an inherited deficiency or lack of proteins involved in metabolic pathways. The majority are defects of single genes that code for enzymes, which catalyze essential biologically and biochemical reactions. Although each specific inborn error of metabolism is rare, taken together, they represent a significant source of congenital disabilities. For instance, it is estimated that a genetically caused inborn error of metabolism occurs in 1/2500 births [1]. A prototypical example of an inborn error of metabolism is homocystinuria due to CBS deficiency [2].

**Figure 1:**
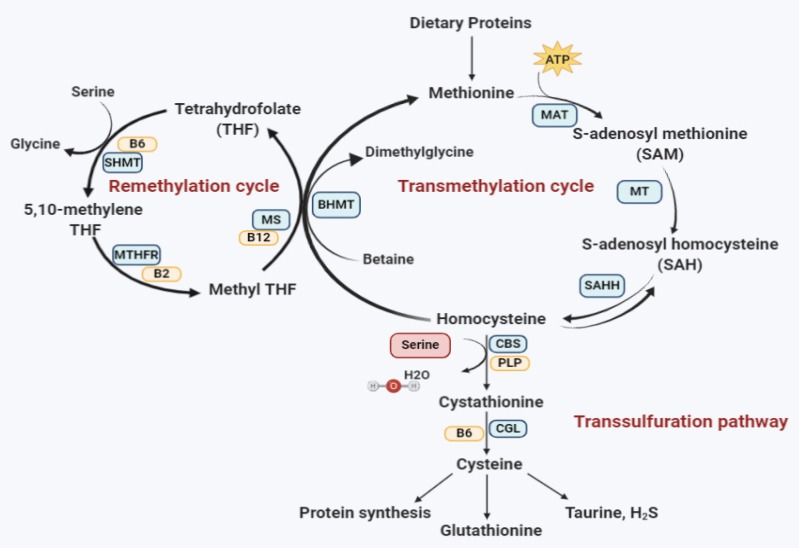
Homocysteine metabolism pathway. Methionine adenosyltransferase (MAT); methyltransferase (MT); S-adenosylhomocysteinase hydrolase (SAHH); betaine-homocysteine methyltransferase (BHMT); pyridoxal-phosphate (PLP); cystathionine gamma-lyase (CGL); methionine synthase (MS); serine-hydroxymethyltransferase (SHMT); methyl-tetrahydrofolate-reductase (MTHFR), hydrogen sulfide (H_2_S).

Classical homocystinuria (OMIM 236200) is an autosomal recessive disease and the most common form of homocystinuria worldwide. It is an inborn error of metabolism with high clinical variability resulting in a metabolic disorder due to deficiency in cystathionine beta-synthase CBS enzyme activity (Figure 1). Consequently, an impaired CBS activity leads to the elevation of both the methionine and homocysteine levels in the blood and urine, leading to hyperhomocysteinemia, homocystinuria, hypermethioninaemia, and hypocysteinaemia. In healthy adults, the concentration of total homocysteine in plasma ranges from 5 to 15 µM; however, it reaches 200 µM in untreated CBS deficient patients [3]. This build-up could lead to several complications such as nearsightedness and dislocated eye lenses. In addition, the raised homocysteine levels could alter the intracellular signaling and cause endoplasmic reticulum stress with endothelial dysfunction [4,5]. Consequently, leading to thromboembolism, vascular, and other diseases [6]. Clinically, CBS deficient patients may present with intellectual disability, a variety of psychiatric and behavioral disorders, skeletal and ocular abnormalities, vaso-occlusive disease, as well as vascular system complications [7]. Affected babies are healthy at birth, but may exhibit a failure to thrive during infancy and developmental delay during the first years of life. To date, there is no cure for homocystinuria and the available treatments target lowering the homocysteine level through betaine, folic acid, and pyridoxine (vitamin B6) supplements. The main aim is to keep the plasma homocysteine as close to the normal range as possible. However, a considerable fraction of affected patients is nonresponsive to pyridoxine treatment. In addition, a methionine-restricted diet is not optimal, and poor compliance leads to serious complications [8]. Therefore, currently the therapeutic approach for homocystinuria is shifted toward stabilizing and rescuing the protein’s native structural conformations through chemical chaperones and proteasome inhibitors. Furthermore, several studies are focusing on other approaches, such as gene and enzyme replacement therapies that will be explained later in Section 6 (Current and potential treatment approaches).

Methionine is an essential sulfur-containing amino acid that is involved in the development and proper mammalian growth. Once the dietary proteins are ingested and methionine enters the cells, methionine adenosyltransferase (MAT) will catalyze the condensation of methionine with ATP and form S-adenosylmethionine (SAM), also known as AdoMet. SAM serves as a crucial methyl donor for various methyltransferase reactions, including hormones biosynthesis and generating S-adenosylhomocysteine (SAH). SAH is then reversibly hydrolyzed into adenosine and homocysteine through the action of S-adenosylhomocysteinase hydrolase (SAHH). Homocysteine is a nonproteinogenic toxic intermediary amino acid residing at the intersections of several pathways. It can be remethylated through methionine synthase (MS) or liver-specific betaine-homocysteine methyltransferase (BHMT), which requires betaine as methyl donors. The other route of homocysteine is the transsulfuration pathway, where cystathionine beta-synthase (CBS) irreversibly converts homocysteine to cysteine. It catalyzes the condensation of homocysteine with serine and forms cystathionine, which is subsequently cleaved by cystathionine gammalyase (CGL) into cysteine and 2-oxobutyrate. Cysteine can be further converted to taurine and hydrogen sulfide (H_2_S). 

Accordingly, the lack of CBS elevates the level of homocysteine and methionine and lowers the concentrations of cystathionine and cysteine in the plasma of homocystinuria patients. It is worth mentioning that both pathways require a particular member of the vitamin B family. For instance, riboflavin (B2) serves as a cofactor for methyl-tetrahydrofolate-reductase, pyridoxine (B6) in serine-hydroxymethyltransferase, CBS and CGL, folic acid (B9) and cobalamin (B12) in methionine synthase. CBS regulates the flow of organic sulfur from methionine to the biosynthesis of all other sulfur-containing compounds such as homocysteine, cysteine, and taurine and their metabolism. Therefore, classical homocystinuria, due to a deficiency in CBS activity, is the most common inborn error of sulfur amino acid metabolism. The prevalence of homocystinuria is around 1/200,000 births worldwide. However, its prevalence in the Gulf region, notably Qatar, is exceptionally high and reached 1:1800 [9].

CBS is a homotetrameric protein with each subunit composed of three structural domains [10,11]: (i) A C-terminal regulatory domain containing the CBS domain tandem and the binding site for the allosteric activator AdoMet (ii) a catalytic core, which is highly conserved and includes the PLP (B6) binding site and; (iii) an N-terminal portion which acts as a heme-binding domain [11,12]. The C-terminal region consists of two CBS domains that are involved in hydrophobic regulatory interactions and could partially shield the active site, thus exhibiting an auto-inhibitory function [13]. SAM binding to the CBS C-terminal domain induces a conformational change that leads to a higher enzymatic activity [14]. This is achieved by eliminating the autoinhibition exerted by the regulatory region, thus, facilitating the access of substrates to the catalytic pocket. In fact, Ereño-Orbea et al. showed that SAM leads to progression of particular CBS mutants toward the activated state. To date, more than 191 pathogenic *CBS* mutations have been documented. The majority of these mutations were identified in Caucasians of European ancestry, whereas only few mutations from African-Americans or Asians were reported [15]. Approximately 87% of all *CBS* mutations are missense and do not target the CBS catalytic site, but rather result in unstable misfolded proteins lacking the normal biological function, designating them for degradation [12]. In addition, a considerable fraction of CBS mutants show impaired response to SAM binding as an allosteric activity modulator and protein stabilizer. This review aims to examine the mutations spectrum of the *CBS* gene in homocystinuria patients with a greater emphasis on those reported in the Middle East and North Africa (MENA) region. 

## 2. The most Common CBS Reported Mutations Worldwide 

For the last three decades, CBS inactivating mutations have been extensively studied in the context of causing homocystinuria [16]. Overall, homocystinuria caused by *CBS* deficiency is considered a relatively rare disease with an incidence rate varying from one in every 200,000 to 335,000 live births. Table 1 summarizes the most common *CBS* mutations that were reported in different parts of the world. Studies showed that CBS is common in some countries, including Ireland (1 in 65,000), Germany (1 in 17,800), Norway (1 in 6400), and reached the highest prevalence in Qatar (1 in 1800) [17]. Homocystinuria is reported as an autosomal recessive disease, where the marriage of two *CBS* carriers’ mutant genes could result in having children with homocystinuria. Furthermore, the high consanguinity rate in the MENA community is considered an important factor that leads to an increase in the prevalence of many metabolic disorders. 

**Figure 2:**
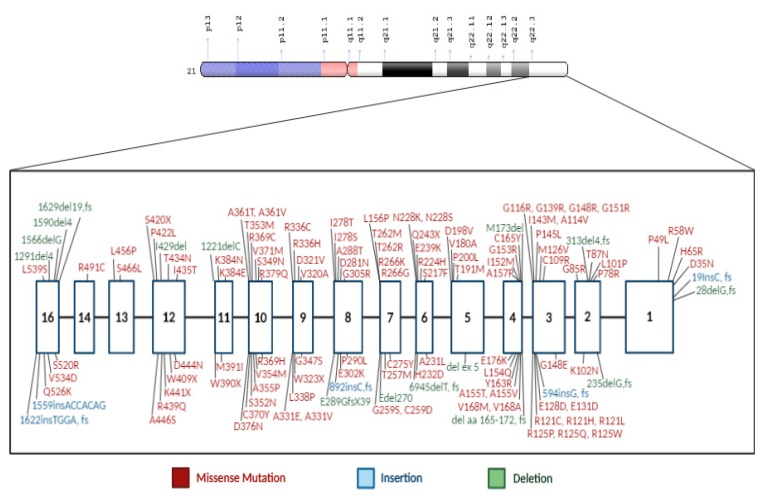
*CBS* gene structure with associated mutations. Exons are represented by white numbered boxes and the *CBS* variants are color coded by mutation type.

The *CBS* gene is located on the long arm of chromosome 21 with 191 variants having been described [40] (Figure 2). The most frequent pathogenic and reported mutations in different countries around the world are p.G307S (31%), and p.I278T (24%) [41,42]. The p.G307S mutation is the most prevalent CBS deficiency mutation in Ireland and Australia [6,23]. It is located on exon 8 of *CBS* gene, where guanine at position 919 is replaced by adenine nucleotide (c.919G>A). This change leads to glycine to serine substitution at position 307. Homozygous patients are severely affected with minimal to nonresponse to pyridoxine (B6) treatment [28,43]. Studies showed that p.G307S mutation is also frequently detected in homocystinuria patients of Celtic descent [43]. Using molecular dynamic simulations, a study showed that p.G307S mutation impaired the catalytic function of the CBS enzyme by preventing the tyrosine residue at position 308 to assume the proper conformational folding. This state is required for forming the pyridoxal–cystathionine intermediate. Additionally, results showed CBS with p.G307S mutation is stable, but inactive, and hence does not respond either to chaperone-based therapy nor pyridoxine treatment [24]. 

Similarly, the p.I278T mutation affects the catalytic domain of the CBS enzyme [16]. Yet, confers responsiveness to pyridoxine treatment [6]. It is considered the most prevalent mutation worldwide, particularly in homocystinuria patients of nonCeltic descent [43]. The p.1278T mutation was first identified in a pyridoxine-responsive patient with mild clinical manifestation [44]. This mutation results from incorrect excision of a 68-bp repeat polymorphism within the *CBS* gene [45]. Consequently, it leads to the substitution of thymine with a cytosine at position 833 (c.883T>C) of exon 8. This change leads to isoleucine to threonine substitution at position 278. The pathogenicity of p.I278T *CBS* mutation was shown to be due to the lack of catalytic activity in a bacterial and yeast expression system. For instance, using the yeast *S. cerevisiae* as an in vitro model showed that p.I278T has only 2.4% of the human wildtype CBS enzymatic activity and failed to complement the growth phenotype [46]. Consequently, patients with p.I278T usually had vascular and connective tissue defects [47]. Other clinical complications of defective CBS enzyme include dislocation of the ocular lenses (ectopia lentis), mental retardation, osteoporosis, and thromboembolism.

## 3. The Prevalence of CBS and Associated Mutations in the MENA Region

The establishment of newborn screening programs has successfully decreased the prevalence of homocystinuria and other aminoacidopathies. Yet, the majority of these disorders arise due to consanguineous marriages [48]. The consanguinity rate reached 40% among first cousins in the Middle East and up to 60% in intermarriages between relatives. Therefore, the incidence of genetic disorders, including homocystinuria, has increased. To date, scarce studies reported the incidence rate of CBS in the MENA region. For instance, the reported incidence in Oman is one in approximately 128,200 births [49], while in Saudi Arabia, the incidence of homocystinuria is two in 100,000 live births [50]. Additionally, in a Kuwait-based study, the frequency of *CBS* mutation among intellectually disabled patients was 0.23% [51]. 

In Qatar, the homozygous mutation c.1006C>T (p.R336C), which is found on exon 9 of the *CBS* gene (Figure 2), a missense mutation in which arginine is replaced by cysteine, consequently resulting in a severe vitamin B6 nonresponsive phenotype [9]. The prevalence of this unique mutation in Qatar is approximately 1:1800 births, where ~6% of the population has a heterozygous p.R336C mutation with an allele frequency of 1% [52]. The p.R336C mutation was first reported in a patient of English descent [28]. Subsequently, it was observed in Australia [53], the Iberian peninsula [54], and recently in Korea [55]. The in vitro expression studies, as well as the enzymatic measurements, along with the clinical symptoms of the patients suffering from this mutation, showed that the mutation eliminated the CBS enzymatic activity. The p.R336C mutation appears to be recurrent in different independent populations. Thus, the correlation between this mutation in the Qatari population and other populations could not be established. Treating the Qatari homocystinuria patients with traditional therapy has been very challenging because the strict low protein content diet is difficult to maintain, and the p.R336C mutation is pyridoxine nonresponsive. Therefore, this disease imposes a financial and clinical burden on the population in Qatar that necessitates an urgent need for the development of an adequate therapy [52,56].

On the other hand, one Qatari patient had a homozygous mutation c.700G>A (p.D234N), which was previously described in Puerto Rican [12] and Venezuelan patients [57]. Similar to c.1006C>T mutation, this mutation includes a hypermutable CpG dinucleotide (transition from guanine to adenine) [58]. According to El-said et al., this mutation has a high chance of recurrence in the Qatari population [59]. However, according to the clinical observation and the genotyping of all Qatari patients suffering from homocystinuria predict the absence of CBS enzymatic activity and nonresponsiveness to vitamin B6 supplements [59]. Although homocystinuria is considered the most prevalent monogenic disease in Qatar, early detection has improved the prevention of some complications, including mental retardation, ectopia lentis, and thromboembolic events [60]. 

In Saudi Arabia, patients suffering from homocystinuria expressed the cardinal biochemical features of homocystinuria. In addition, most of the clinical manifestations reported due to homocystinuria in the Saudi Arabian cohort were related to the ophthalmological, musculoskeletal, vascular, and nervous systems complications [7,27,61]. For instance, some Saudi patients, who suffered from white matter abnormalities, were also reported with homocystinuria. However, Zaidi et al., reported a case of Legg–Calv´e–Perthes disease, a previously unknown complication in homocystinuria, which was observed in one patient suffering from a novel p.W323X mutation in the *CBS* gene [62]. Zaidi et al., explained the occurrence of Legg–Calv´e–Perthes disease as vascular thromboembolism due to the persistently elevated homocysteine leading to vascular necrosis of the femoral head in their patients. This novel mutation is considered the predominant mutation in Saudi Arabia and was reported in 10 Saudi families. This could be due to the high consanguinity marriage causing homozygosity of the recessive alleles and the expression of disease phenotype in patients. Similarly, two recurrent mutations, c.457G>A (p.G153R) and c.1006C>T (p.A336C), were reported in two Saudi families and described previously [28,53,63].

In Palestinian Arab homocystinuria patients, six different *CBS* mutations, c.304A>C, c.833T>C, c.785C>G, g.1627del19, IVS17 (g18327del 5), and IVS4 (g6643del 29), have been reported in this highly inbred population [64]. These mutations were novel and were not reported in the neighboring regions. However, patients living in the same villages carry the same genetic alterations, thus indicating a founder effect. Despite the adjacent geographic region of Palestine, Saudi, Palestinian Arabs have a different spectrum of the *CBS* gene mutations. The narrow geographic spread of these mutations suggests that they arose over the past few generations [64].

In Sudan, a *CBS* mutation c.770C>T (p.T257M or pThr257Met) was reported. Interestingly, the same mutation was also reported in Spanish and Italian families (15,17). The pThr257Met mutation was shown to have <1% of the CBS enzyme activity and conferred nonresponsiveness to pyridoxine treatment. Table 2 summarizes the identified *CBS* mutations that were reported in different countries of the MENA region.

## 4. Recent Novel CBS Mutations 

Recently, two novel mutations, a missense change (c.467T>C; p.L156P) and an in-frame deletion (c.808_810del; p.E270del) were reported in Pakistani children [65]. The mutations were identified using Sanger sequencing and resulted in hyperhomocysteinemia and lens dislocation in three patients from different families. In addition, eight novel mutations in *CBS* were identified in Chinese patients with classical homocystinuria. None of these missense mutations were reported in other regions previously. Moreover, the effect of these mutations on *CBS* gene expression, CBS protein expression, stability, and activity is not investigated yet. However, Li et al., with the use of the PolyPhen-2 prediction software, indicated that the p.T236A and p.L230G are probably damaging while the p.L72I is a benign mutation. On the other hand, the SIFT prediction software indicated that the p.T236A and p.L230G are damaging, while the p.L72I is a tolerated mutation (17). Similarly, a total of 35 samples were obtained from Brazilian patients with a biochemically confirmed diagnosis of classical homocystinuria [42]. Eight novel mutations [c.2T>C, c.209+1delG, c.284T>C, c.329A>T, c.444delG, c.864_868delGAG, c.989_991delAGG, and c.1223+5G>T] were found. It is worth mentioning that there was high variability in the genotypes, and most of the patients in this study were pyridoxine nonresponsive. Generally, the treatment of homocystinuria patients with varying missense mutations is based on a combined high-dose of cofactors of homocysteine metabolism and a life-long methionine-restricted diet. Yet, less than 50% of affected individuals show a significant reduction in plasma homocysteine levels after treatment [7,66].

## 5. Clinical Diagnosis and Disease Detection

Clinically, patients with classic homocystinuria exhibit numerous manifestations during infancy because homocysteine disrupts the development of many organ systems. These include Marfanoid habitus, pectus exavatum (curved-in sternum), pectus carinatum (protruding sternum), genu valgum, where knees are angled toward each other, and many other skeletal deformities [67,68]. In addition, ectopia lentis (dislocation of the lens) can lead to nearsightedness and blurred vision. Homocystinuria was first discovered in 1962 in Northern Ireland, where the patients had mental retardation [69]. Shortly after that, CBS enzyme deficiency was demonstrated as the causative factor for this abnormality [70]. Significant progress has been achieved in diagnosing homocystinuria since the time of its discovery. 

**Table 2:** *CBS* mutations and clinical phenotypes of homocystinuria in Arab countries.

Country	Nucleotide Change	Protein Change	Exon	Mutation Type	Consanguinity	Pathogenicity and most Common Associated Phenotype Phenotypes	Responsive to Pyridoxine (Vitamin B6) Treatment	Reference
Qatar	c.1006C > T	p.R336C	11	Missense	-	Pathogenic-Hyperhomocystimia, Disproportionate tall stature, Lens luxation, Thromboembolism, Intellectual disability Seizures	Nonresponsiveness	[9,52,59,67]
					Yes (98.4% of the patients)			[68]
					Yes (84% of the patients)			[71]
	c.700G>A	p.D234N	8	Missense	Yes	Pathogenic-Skeletal and ocular abnormalities	Nonresponsiveness	
	c.1039G>A	p.G347S	9	Missense	-	-	Not known	
Saudi Arabia	c.969G>A	p.Trp323X	9	Novel nonsense mutation	Yes	Pathogenic-a truncation of the CBS protein and results in the complete loss of the CBS enzyme activity. Elevated plasma levels of homocysteine and methionine	Nonresponsiveness	[42,72]
	c.1006C>T	p.R336C	11	Missense	Yes	Pathogenic-Hyperhomocystimia, Disproportionate tall stature, Lens luxation, Thromboembolism, Intellectual disability Seizures	Nonresponsiveness	[72]
	c.457G>A	p.G153R	4	Missense	Yes	Likely pathogenic	-	[72]
Oman	844ins68	-	8	Insertion	-	Aneurysmal subarachnoid hemorrhage	-	[73]
Lebanon	c.1152 G>C	p.K384N	11	Missense	-	-	-	Unpublished data
Sudan	c.770C>T	p.T257M	7	Missense	Yes	Pathogenic due to complete loss of CBS enzyme activity	Nonresponsiveness	[55,72]
Palestinian Arab	c.304A>C	p.K102Q	2	Missense	Yes	Likely benign-Ectopia Lentis, Marphanoid features, Thromboembolic episodes	Variable	[64]
	c.833T>C	p.I278T	8	Missense		Pathogenic-Ectopia lentis, Marphanoid feature, mental retardation, idiopathic infertility	Responsive	
	c.785C>G	p.T262R	7	Missense		Pathogenic-Thromboembolic episodes, mental retardation	-	
	g.1627del 19	addition of non-coded 17 amino acids	16	Deletion/ Frameshift		Pathogenic	-	
	IVS17 (g18327del 5).	-	16	Deletion		Pathogenic-Ectopia lentis, mental retardation	-	
	IVS4 (g6643del 29)	29 bp deletion (exon 5 skipping)	5	Deletion		Pathogenic	-	
Pakistan	c.467T>C	p.L156P	7	Missense	Yes	Pathogenic-Developmental and Neurological problems. Myopia and lens dislocation	-	[65]
	c.808_810del	p.E270del	10	In-frame deletion	Yes	Pathogenic Developmental and Neurological problems. Glaucoma and subluxated lens.	-	

Although the management and clinical diagnosis of homocystinuria could differ from country to country, this disorder is mostly diagnosed based on the patient clinical features and routine metabolic testing. The analysis of plasma and urine amino acid will show a frequent elevation of both methionine (>50 µmol/L) and homocysteine (>100 µmol/L). Some Arab countries have implemented the utilization of several molecular diagnostic techniques, including mass spectrometry (MS), high-pressure liquid phase chromatography (HPLC), and sequencing techniques such as Sanger and next-generation sequencing [74]. In addition, molecular genetic testing and neonatal screening programs have been incorporated in the disease screening system as early intervention could facilitate effective treatment. Yet, this genetic monitoring requires high-throughput, reliable, and rapid methods to extract DNA and subsequently analyze the mutations, which may not be universally available and could be challenging to implement [52]. Additionally, some technologies are currently emerging in rapid metabolite-based disease biomarker screening. For instance, a recent study identified paper spray ionization mass spectrometry (PSI-MS) for analyzing metabolic biomarkers associated with children disorder [75]. As mass spectrometry is a recognized gold standard technique for an inborn error of metabolism study, PSI-MS shows great potential for point-of-care clinical applications. Additionally, PSI-MS could be a great tool for early stage rapid biomarker discovery for Homocystinuria. In addition, other assays depend on measuring the CBS activity through a fluorescent probe. For instance, the assay utilizes homocysteine and cysteine as a substrate to produce hydrogen sulfide. The latter will react with a probe containing azido-functional group and consequently yielding a fluorescent amino group. Previously, an elevated level of methionine was detected through screening of homocystinuria biochemical metabolites. However, this method had poor sensitivity. In addition, it was technically difficult to quantify homocysteine in dried blood spots (DBS). Due to the high prevalence of homocystinuria and, because early detection could significantly benefit children, a dual strategy screening has been developed in Qatar to screen all newborns with homocystinuria [58]. The national biochemical and molecular newborn screening (NBS) program has been established in Qatar in 2006, which includes both biochemical and molecular screening approaches. These include combining HPLC with tandem mass spectrometry to quantify the total homocysteine in DBS. Additionally, rapid high-throughput genetic screening has been introduced to detect *CBS* gene mutations that are identified in Qatari population including p.R336C and p.D234N. This was achieved by utilizing an automated 96-well plate DNA extraction method followed by individual PCR-based fluorogenic TaqMan probe assays [58].

Apart from the disease management and complications that may arise during the patient’s lifespan, parents should be aware that there is a 25% chance of recurrence risk in a subsequent pregnancy. Additionally, there is an increased likelihood that a child with homocystinuria may have siblings who are potential carriers [41]. Therefore, genetic counseling is recommended for prospective parents, especially those with a history of homocystinuria in the family. Moreover, prenatal diagnosis can be implemented and achieved through culturing the chorionic villi and amniotic cells to test for the presence of CBS enzyme [76].

## 6. Current and Potential Treatment Approaches 

The early detection of homocystinuria along low protein and methionine restricted diet is the best treatment approach for all types of homocystinuria patients [68]. Referring to the homocystinuria metabolism pathway (Figure 1), the administration of folic acid and pyridoxine is also considered as an effective therapy, especially in pyridoxine-responsive patients, as these supplements have shown to enhance the CBS enzyme residual activity in more than half of the patients [77]. Yet, they were not effective in patients with B6 nonresponsive mutations such as the p.R336C [58]. Although significant advances were made toward curing homocystinuria and protein misfolding, yet, there are no curative treatments for this disease. Protein misfolding and its accumulation are the upstream events in the pathological cascade. Thus, stabilizing and rescuing the protein’s native structural conformations is a targeted therapeutic approach. 

Recently, chaperones have been recognized as effective molecules in reducing the accumulation of misfolded proteins. Accordingly, minimizing their downstream pathological consequences. Several chemical chaperones are receiving attention as possible treatments for many misfolded protein mutations. For instance, the administration of 2% of the trehalose solution orally increased the stability of the huntingtin containing protein and improved the motor dysfunction in a mouse model [78]. Randomized clinical trials proved that an FDA-approved Buphenyl, used for treating urea cycle disorders, causes substantial stimulation of chloride transport in homozygous patients for F508 CFTR. Thus, it is a viable therapeutic approach for cystic fibrosis patients [79]. 

In regards to CBS, Kopecka et al. studied in vitro the effect of selected chaperones including δ-aminolevulinic acid (δ-ALA) and three osmolytes (taurine, betaine, and glycerol) on 27 different CBS mutants. Of which, 14 mutants responded by at least 30% increase in the enzymatic activity, and four mutants showed an increased formation of CBS tetramers, without an increase in the CBS activity [80]. For instance, PBA and TUDCA are FDA approved chemical chaperones used for urea cycle disorders and biliary cirrhosis treatment, respectively [81]. Furthermore, studies showed that PBA attenuates the endoplasmic reticulum (ER) stress and acts as an ammonia scavenger in urea cycle disorders [82]. Similarly, TUDCA has been shown to mitigate the ER stress and have an anti-apoptotic and antioxidant activity [83]. 

Another potential chemical chaperone is betaine, which uses an alternative pathway to convert homocysteine into methionine, and was reported to be effective in pyridoxine nonresponsive patients [84]. In an attempt to rescue the functional defect of the p.R336C CBS mutation, a study was conducted using knock-in p.R336C mutation in HEK293T cell line [9]. Cells were treated with different types of chaperones (betaine, glycerol, sorbitol, and proline) at different concentrations. Interestingly, betaine was able to restore the structural defect of the p.R336C protein in vivo, but not the activity of the p.R336C mutation. Various treatment approaches, such as chemical chaperones [9], CBS enzyme replacement therapy, and gene therapy [85] could be promising alternative treatments for homocystinuria patients. Additional treatment strategies including the administration of proteasome inhibitors or protein stabilizing drugs to prevent early degradation of mutated CBS protein. For example, a recent report showed that proteasome inhibitors rescued the phenotype in p.I278T and p.S466L mutant mice [86]. Furthermore, it was documented that bortezomib was able to restore both the growth and CBS activity in p.I278T mutant yeast and mice [87]. Other potential chemical chaperones include tauroursodeoxycholic acid (TUDCA), sodium 4-phenylbutyrate (PBA), urea derivatives chemical chaperones such as trimethylamine oxide (TMAO), amino acid derivatives chemical chaperones such as glycine, polyols chemical chaperones such as glycerol and sorbitol, mannitol, maltose, phenyl butyric acid. Additionally, the use of aminothiols, such as cysteamine, holds potential clinical promise to target arginine to cysteine mutations of different genetic disorders [88]. It is worth mentioning that SAM analogs are also considered pharmacological chaperones and kinetic stabilizers that could prevent the misfolding and rapid degradation of mutant CBS protein [89]. Studies demonstrated that CBS tetramer is stabilized by SAM binding to a site different from the allosteric site responsible for activation, and presented natural analogues that could stabilize CBS tetramer without interfering with allosteric activation. Thus, rescuing the CBS activity in homocystinuria patients [89,90]. Yet, some mutations, such as R125Q, E176K, P422L, and S466L, are SAM-nonresponsive mutants [90].

Shifting to in vivo models, a study previously described the development of a genetically engineered mouse that expresses the p.R336C human CBS protein as its only source of CBS (Tg-R336C Cbs-/-) [91]. As expected, these mice have extreme elevation in both serum total homocystinuria and liver total homocystinuria compared to control transgenic mice. However, the administration of bortezomib showed a complete rescue of CBS activity in these mice. The creation of such an in vivo system to modulate plasma homocysteine would be useful in the study of homocysteine-related diseases and finding novel therapeutic approaches for CBS deficiency. For instance, a study used a minicircle-based naked DNA gene therapy technique to treat CBS deficient mice (Tg-I278T Cbs-/-) [92]. Following the injection of mice with the DNA-minicircle vector, results showed a significant decrease in serum total homocysteine level with a 34-fold increase in liver CBS activity. Therefore, the study suggested that minicircle-based gene therapy could be a potential treatment for CBS deficiency. Another promising approach is the enzyme replacement therapy. It involves intravenous infusions to correct the deficiency or absence of an enzyme in the body. This approach was reported to be successful in lysosomal storage diseases such as Gaucher disease, Fabry disease, as well as severe combined immunodeficiency [86]. However, the use of enzyme replacement therapy to treat homocystinuria due to CBS deficiency is not well addressed. Therefore, a recent study aimed to address the core enzyme deficiency through the long-term administration of PEG-CBS [8]. Results showed that enzyme replacement therapy significantly reduced the plasma homocysteine concentration and normalized plasma cysteine for up to nine months of treatment. The metabolic and biochemical balance was restored and improved in the kidney, liver, and brain of the CBS deficient mice (Tg-I278T Cbs-/-). Therefore, enzyme replacement therapy has the potential to successfully correct the clinical manifestations in homocystinuria patients and improve their life quality. 

## 7. Conclusions

Homocystinuria is a monogenic disease caused by a deficiency in the activity of the CBS enzyme. This study highlighted the necessity for further epidemiological studies in Arab countries, especially in areas with high prevalence of consanguineous marriage. High consanguinity rate in the MENA community is considered a factor that could also increase the prevalence of many metabolic disorders including homocystinuria. Therefore, public health strategies should be implemented to increase public understanding and awareness of the consequent genetic risks of consanguineous marriages. In addition, screening programs should be applied in all Arab nations to facilitate its early detection and consequent treatment. Although significant advances have been made toward curing homocystinuria, yet, there are no curative treatments for this disease. Chemical chaperones and protein stabilizers are considered potential therapeutic approaches for stabilizing and rescuing the protein’s native conformations. Thus, restoring the CBS enzyme activity.

## Figures and Tables

**Table 1:** Cystathionine beta-synthase (*CBS)* mutations and clinical phenotypes of homocystinuria reported worldwide.

Country	Nucleotide Change	Protein Change	Exon	Mutation Type	Consanguinity	Pathogenicity and most Common Associated Phenotype Phenotypes	Responsive to Pyridoxine (Vitamin B6) Treatment	Reference
Western Eurasians	c.833T>C	p.I278T	8	Missense	No	Pathogenic-Ectopia lentis, Marphanoid feature, mental retardation, idiopathic infertility, severe vascular complications	Responsiveness	[18]
Denmark Norway Germany Czech and Slovak Republics	c.833T>C	p.I278T	8	Missense	-	Pathogenic-Ectopia lentis, Marphanoid feature, mental retardation, idiopathic infertility, severe vascular complications	Responsiveness	[19,20]
Czech	c.1105C>T	p.R369C	10	Missense	-	Patients do not have any clinical symptoms at all. The late onset of symptoms, lack of mental retardation, and connective tissue involvement	Responsiveness	[21]
Slovakia	526 G>A	p.E176K	4	Missense	No	Conflicting interpretations of pathogenicity. Thoracic aortic aneurysm and aortic dissection. Cardiovascular phenotype	Nonresponsiveness	[22]
	1226 G>A	p.W409X	12	Nonsense	No	-	Nonresponsiveness	
Netherland	c.833T>C	p.I278T	8	Missense	No	Pathogenic-Ectopia lentis, Marphanoid feature, mental retardation, idiopathic infertility, severe vascular complications	Responsiveness	[12,19]
	c.539 T>C	p.V180A	5	Missense	No	-	-	[12]
	c.1330G>A	p.D444N	12	Missense	No	Mild	-	
Ireland	c.919 G>A	p.G307S	8	Missense	-	Pathogenic-Cardiovascular phenotype	Nonresponsiveness	[23,24,25]
	c.306 G>C	p.K102N	2	Missense	No	Likely pathogenic-Neurogenic bladder or bowel signs, developmental delay, mental retardation	Responsiveness	[26]
Italy	c.146 C>T	p.P49L	1	Missense	No	Pathogenic, VUS Cardiovascular phenotype	Responsiveness	[27]
	c.172 C>T	p.R58W	1	Missense	No	Mild mental retardation with EEG anomalies, osteoporosis, malar flush, and ultrasound evidence of arterial disease	Nonresponsiveness	[28]
	c.262 C>T	p.P88S	2	Missense	-	Pathogenic-Zonular pulverulent cataract phenotype	-	[29]
	c.469G<C	p.A157P	4	Missense	No	Pathogenic-Pectus escavatum, Tricuspid valve prolapse, Kyphoscoliosis, Iridodonesis, Mild elbow valgus	-	[30]
Spain	c.869 C>T	p.P290L	8	Missense	No	Hermansky Pudlak syndrome 2	Responsiveness	[29]
	c.572C>T	p.T191M	5	Missense	No	Pathogenic	Nonresponsiveness	[31]
	c.833T<G	p.I278S	8	Missense	No	Pathogenic	Responsiveness	[32]
Portugal	c.572C>T	p.T191M	5	Missense	No	Pathogenic	Nonresponsiveness	[31]
Venezuela	c.700G>A	p.D234N	8	Missense	Yes	Pathogenic-Skeletal and ocular abnormalities	Nonresponsiveness	[33]
France	1150 A>G	p.K384E	11	Missense	No	Pathogenic	Responsiveness	[34]
	1616 T>C	p.L539S	16	Missense	No	Pathogenic	Responsiveness	
UK	c.374G>A	p.R125Q	3	Missense	No	Pathogenic Cardiovascular phenotype	-	[35]
	c.430G>A	p.E144K	3	Missense	No	Pathogenic Cardiovascular phenotype	-	[35]
	c.833T>C	p.I278T	8	Missense	No	Pathogenic-Ectopia lentis, Marphanoid feature, mental retardation, idiopathic infertility, severe vascular complications	Responsiveness	[35]
	c.919G>A	p.G307S	8	Missense	No	Pathogenic-Cardiovascular phenotype	Nonresponsiveness	[25,35]
USA	c.341C>T	p.A114V	3	Missense	No	-	-	[35]
	c.374G>A	p.R125Q	3	Missense	No	Pathogenic-Cardiovascular phenotype	Nonresponsiveness	[35,36]
	c.785C>T	p.T262M	7	Missense	No	Pathogenic-Thromboembolic episodes, mental retardation	Nonresponsiveness	[35,36]
	c.797G>A	p.R266K	7	Missense	No	-	Responsiveness	[35]
	c.833T>C	p.I278T	8	Missense	No	Pathogenic-Severe vascular complications	Responsiveness	[35]
	c.919G>A	p.G307S	8	Missense	No	Pathogenic-Cardiovascular phenotype	Nonresponsiveness	[25,35]
	g.13217A>C	(del ex 12)	Intron 11	Deletion	No	-	-	[35]
	c.1330G>A	p.D444N	12	Missense	No	Pathogenic-Psychomotoric retardation and marfanoid features	Partially pyridoxine-responsive	[35,37]
India	c.518delTGA	p.M173del	4	Deletion	No	Pathogenic	-	[32]
Argentina	c.676G<A	p.A226T	6	Missense	No	Mild-Hypertrophic cardiomyopathy	Responsiveness	[25,32]
	c.962A<T	p.D321V	9	Missense	No	Pathogenic-Crouzon syndrome	-	[32,38]
	c.1336G<T	p. A446S	12	Missense	No	Mild-lens dislocation	Responsiveness	[32]
Australia	c.833T>C	p.I278T	8	Missense	No	Pathogenic-Ectopia lentis, Marphanoid feature, mental retardation, idiopathic infertility, severe vascular complications	Responsiveness	[39]
